# Ceftriaxone-induced toxic epidermal necrolysis mimicking burn injury: a case report

**DOI:** 10.1186/1752-1947-3-9323

**Published:** 2009-12-10

**Authors:** Sarit Cohen, Allan Billig, Dean Ad-El

**Affiliations:** 1 Department of Plastic Surgery, Rabin Medical Center, Beilinson Hospital, Petah Tiqwa, Israel; 2 Sackler Faculty of Medicine, Tel Aviv University, Tel Aviv, Israel

## Abstract

**Introduction:**

Toxic epidermal necrolysis is a rare exfoliative disorder with a high mortality rate.

**Case presentation:**

We present a 70-year-old woman of Iranian descent who presented with toxic epidermal necrolysis that was initially diagnosed as a scald burn. Further anamnesis prompted by spread of the lesions during hospitalization revealed that the patient had been receiving ceftriaxone for several days. To the best of our knowledge, this is the first case of ceftriaxone-induced toxic epidermal necrolysis in the English literature.

**Conclusion:**

Toxic epidermal necrolysis is an acute, life-threatening, exfoliative disorder with a high mortality rate. High clinical suspicion, prompt recognition, and initiation of supportive care is mandatory. Thorough investigation of the pathogenetic mechanisms is fundamental. Optimal treatment guidelines are still unavailable.

## Introduction

Toxic epidermal necrolysis(TEN) is a rare, potentially life-threatening disorder characterized by widespread epidermal death [1,2]. The majority of reported cases were the result of idiosyncratic drug reactions [3]. The severity of the syndrome, the anecdotal case reports, and the uncontrolled series presented in the English literature render accurate characterization of the syndrome difficult in terms of underlying pathogenic mechanisms and adequate treatment options.

We present a case of TEN diagnosed initially as a scald burn. The similar initial dermatological manifestations of these entities might be confusing to the clinician, especially when the patient is disoriented and an accurate anamnesis is difficult to obtain.

In the present case, TEN was caused by ceftriaxone therapy. To the best of our knowledge, this is the first case of ceftriaxone-induced TEN in the English literature.

## Case presentation

A 70-year-old woman of Iranian descent was referred to our trauma unit for a major scald burn. The exact mechanism of injury was inconclusive. The patient had a history of diabetes mellitus type 2, ischemic heart disease, hypertension, hyperparathyroidism, hyperlipidemia, chronic bronchitis, glaucoma, and mild depressive disorder. She had been receiving treatment on a regular basis with the following medications: amitriptyline, enalapril, glyburide, verapamil, omeprazole, aspirin, simvastatin, theophylline, furosemide, metformin, citropram, dorzolamide hydrochloride eye drops, and latanoprost eye drops.

**Figure 1:**
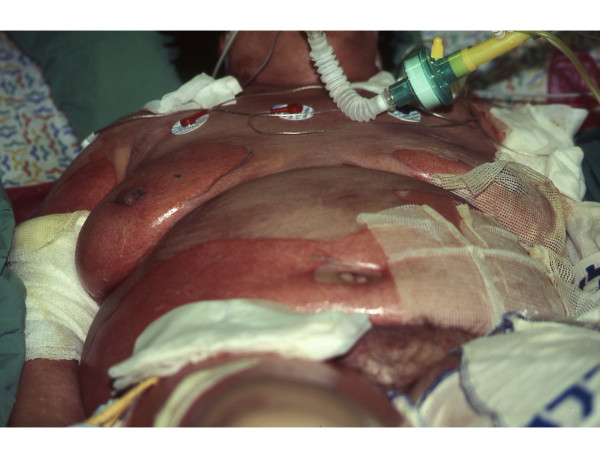
**Clinical manifestation of TEN, demonstrating widespread epidemiolysis affecting bilateral breast, lower abdomen, and anteromedial aspect of the right arm**. The central anterior trunk is not affected.

**Figure 2:**
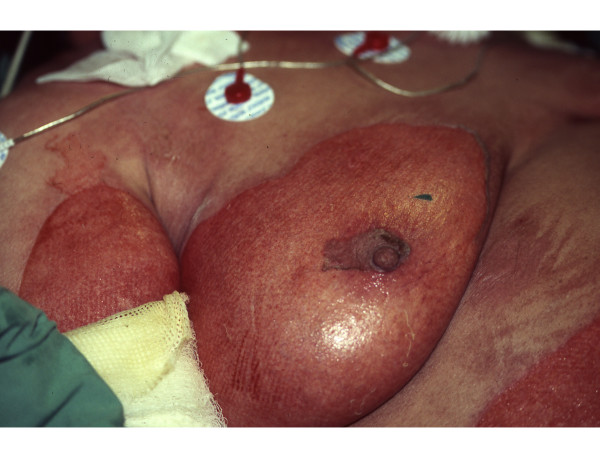
**Closer view demonstrating the epidermolysis in the right breast**.

On admission, the patient was disoriented. Blood pressure was 90/60 mmHg. Cutaneous examination revealed a second-degree superficial burn involving both breasts, lateral aspect of the flanks, anteromedial aspect of the arms, medial aspect of the thighs, and the right scapular region. Diffuse erythema was noted, especially of the upper extremity and anterior trunk (Figs. 1,2).

A presumptive diagnosis of a second-degree, superficial major scald burn affecting 26% of the total body surface area (TBSA) was made. Fluid resuscitation was initiated according to the Parkland formula [4]. A Foley catheter was inserted. Local treatment included wound debridement and application of saline-soaked gauze.

Physician examination 12 hours post-admission to the Burn Unit was remarkable for thin blisters in locations not affected on admission: back, neck, inguinal region, and both knees (Figs. 1, 2), ultimately effecting 35% of the TBSA. The worsened epidermolysis was accompanied by a positive Nikolsky sign.

On further questioning, burn was ruled out as a causal factor. The patient reported that 2 days prior to admission, she had been discharged from another hospital with a diagnosis of pneumonia, and she had been receiving ceftriaxone for 4 days.

The final diagnosis was TEN due to ceftriaxone intake. The mucous membranes were not involved. Treatment with intravenous hydrocortisone 500 mg was initiated. The hypoglycemia (glucose level-45 mg/dl) was successfully treated with intravenous dextrose 5%, and the oral hypoglycemic medications were discontinued. Laboratory studies revealed hypomagnesemia (1.32 mg/dl), for which intravenous MgS0_4 _was administered. Local treatment included Vaseline gauze dressings that were changed once a day.

**Figure 3:**
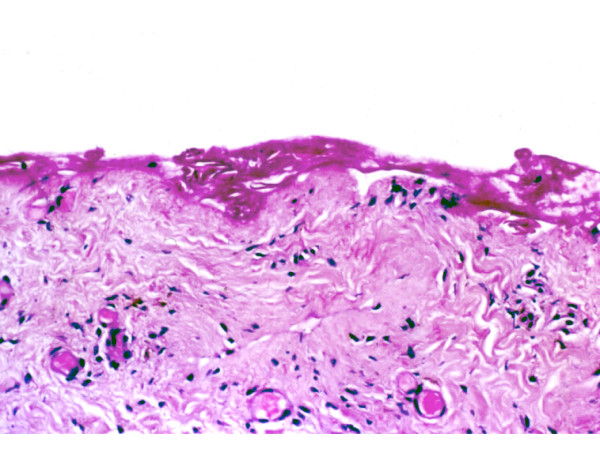
**Skin biopsy demonstrating full thickness epidermal necrosis**. The dermis is devoid of inflammatory cells. (H&E, original magnification ×200).

On the second day of admission, the patient's temperature began to rise. Complete blood count revealed leukopenia of 2,300 mg/dl. Incisional punch biopsy demonstrated widespread full-thickness epidermal necrosis (Fig. 3). The dermis was devoid of inflammatory cells. Histopathological findings were compatible with the diagnosis of TEN. The patient was referred to our intensive care unit (ICU), and treatment with intravenous immunoglobulins (IVIG) was initiated (0.5 g/kg daily for 4 days, the total daily dose of IVIG was 40 grams). The hemodynamic instability was successfully treated with inotropic agents and mechanical ventilation.

Blood culture results, obtained during the patient's hospitalization in the ICU, were positive for *Klebsiella pneumoniae, Proteus mirabilis, Enterobacter, Enterococcus *and *Pseudomonas aeruginosa*. Antibiotic treatment included vancomycin, levofloxacin, ciprofloxacin, ampicillin sulbactam, piperacillin tazobactam, and amikacin sulfate. The clinical course was complicated by adult respiratory distress syndrome, thrombocytopenia, and hypoglycemic episodes. Following prolonged ventilation, tracheostomy was performed. After 42 days in the ICU, the patient was found to be hemodynamically stable and afebrile, and was discharged to rehabilitation. Study of the cutaneous lesions demonstrated re-epithelization with successful wound healing. Mild pigmentary alterations remained with no residual scars. Despite the favorable course of TEN in this case, the patient succumbed to intracranial hemorrhage 4 months later. This outcome was entirely unrelated to TEN.

## Discussion

TEN is a rare exfoliative disorder with an estimated annual incidence of 1-2 per million [2]. Reported mortality rates vary from 20 to 60 percent [3]. The most common cause of TEN is idiosyncratic drug reaction, although viral, bacterial, and fungal infections, as well as immunization, have been described [3]. The drugs most frequently involved are nonsteroidal anti-inflammatory agents, chemotherapeutic agents, antibiotics, and anticonvulsants [3,5]. Among the cephalosporins, ceftazidime [6], cefuroxime [7], cephalexin [7-10], and cephem [11] have been implicated. To the best of our knowledge, this is the first reported case of TEN induced by ceftriaxone.

The pathogenesis of TEN is still not fully clear. The widespread epidermal death is thought to be a consequence of keratinocyte apoptosis [12]. A pivotal role of cytotoxic T lymphocytes has been suggested [3]. Recent studies indicated that TEN may be an MHC-class -I-restricted specific drug sensitivity resulting in clonal expansion of CD8+ cytotoxic lymphocytes with potential for cytolysis. The cytotoxicity is apparently mediated by granzymes (serine proteinases that are components of cytotoxic cells and natural killer cell granules) [13].

The clinical course of TEN is characterized by a prodromal phase with influenza-like symptoms followed by intense erythema, urticarial plaques, and bullae which progress over a day or two to a more generalized epidermal slough [3]. There is often severe involvement of the mucosal surfaces that may precede the skin lesions. Functionally, mucosal involvement might entail impaired alimentation and higher vulnerability to infections, rendering the prognosis less favorable. As such, the absence of mucosal involvement in the case presented may have contributed to her favorable outcome. Progressive neutropenia and thrombocytopenia may develop within a few days and, together with septic complications, may lead to multi-organ failure and death. Apart from prompt withdrawal of the causative drug and rapid initiation of supportive care, strict therapeutic guidelines are still lacking. The benefit of individual treatment options is a matter of debate, and the reader is referred to the comprehensive review by Chave et al. [1]

Despite the controversial efficacy of intravenous immunoglobulins and corticosteroids, our patient was treated with both. We do not know, however, which agent was responsible for her clinical improvement.

## Conclusion

TEN is an acute, life-threatening, exfoliative disorder with a high mortality rate. High clinical suspicion, prompt recognition, and initiation of supportive care is mandatory. Thorough investigation of the pathogenetic mechanisms is fundamental. Optimal treatment guidelines are still unavailable. Multi-institutional collaborative efforts to develop better treatment strategies are warranted.

## Abbreviations

TEN: toxic epidermal necrolysis; ICU: intensive care unit.

## Consent

Consent from the patient herself was not possible due to her unexpected death. As per consent from next of kin, this patient was childless and had no first degree relatives in the country (she immigrated from Iran on her own in 1951)

We refrained from using any identifying patient characteristic (written or visual), thereby fully respecting her confidentiality.

## Competing interests

The authors declare that they have no competing interests.

## Authors' contributions

SC collected the data and wrote the report, and was involved in drafting the manuscript. AB was involved in drafting the manuscript. DA-E revised the manuscript critically for important intellectual content. All authors read and approved the final manuscript.

## References

[B1] Chave TA Mortimer NJ Sladden MJ Hall AP Hutchinson PE Toxic epidermal necrolysis: current evidence, practical management and future directions Br J Dermatol 2005 153 241 253 10.1111/j.1365-2133.2005.06721.x 16086734

[B2] Ducic I Shalom A Rising W Nagamoto K Munster AM Outcome of patients with toxic epidermal necrolysis syndrome Plast Reconstr Surg 2002 110 3 768 773 10.1097/00006534-200209010-00008 12172137

[B3] Avakian R Flowers FP Araujo OE Ramos-Caro FA Toxic epidermal necrolysis: A review J Am Acad Dermatol 1991 25 1 part 1 69 79 10.1016/0190-9622(91)70176-3 1880257

[B4] Scheulen JJ Munster AM The Parkland formula in patients with burns and inhalation injury J Trauma 1982 22 10 869 871 10.1097/00005373-198210000-00011 7131606

[B5] Baroni A Ruocco E Lyell syndrome Skin Med 2005 4 4 221 225 10.1111/j.1540-9740.2005.03593.x 16015071

[B6] Thestrup-Pedersen K Hainau B Al'Eisa A Al'Fadley A Hamadah I Fatal toxic epidermal necrolysis associated with ceftazidime and vancomycin therapy: a report of two cases Acta Derm Venereol 2000 80 4 316 317 10.1080/000155500750012342 11028879

[B7] Yossepowitch O Amir G Safadi R Lossos I Ischemic hepatitis associated with toxic epidermal necrolysis in a cirrhotic patient treated with cefuroxime Eur J Med Res 1997 2 4 182 184 9110927

[B8] Dave J Heathcock R Fenelon L Bihari DJ Simmons NA Cephalexin induced toxic epidermal necrolysis J Antimicrob Chemother 1999 28 3 477 478 10.1093/jac/28.3.477 1960132

[B9] Hogan DJ Rooney ME Toxic epidermal necrolysis due to cephalexin J Am Acad Dermatol 1987 17 5 pt 1 852 853 10.1016/S0190-9622(87)80290-6 3680666

[B10] Jick H Derby LE A large population based follow-up study of trimethoprim-sulfamethoxazole, trimethoprim, and cephalexin for uncommon serious drug toxicity Pharmacotherapy 1995 15 4 428 432 7479194

[B11] Okana M Kitano O Ohzono K Toxic epidermal necrolysis due to cephem Int J Dermatol 1988 27 3 183 184 10.1111/j.1365-4362.1988.tb04928.x 3372117

[B12] Paul C Wolkenstein P Adle H Wechsler J Garchon HJ Revuz J Roujeau JC Apoptosis as a mechanism of keratinocyte death in toxic epidermal necrolysis Br J Dermatol 1996 134 710 714 10.1111/j.1365-2133.1996.tb06976.x 8733377

[B13] Miyauchi H Hosokawa H Akaeda T Iba H Asada Y T cells subsets in drug-induced toxic epidermal necrolysis. Possible pathogenic mechanism induced by CD8-positive T cell Arch Dermatol 1991 127 851 855 10.1001/archderm.127.6.851 2036032

